# Organized and Fugitive VOC Emissions from Typical Industrial Parks and Their Impact on Secondary Pollution

**DOI:** 10.3390/toxics14030242

**Published:** 2026-03-10

**Authors:** Tao Liu, Xiaoning Li, Weidong Wu, Min Yan, Yanxin He, Xudong Quan, Peng Liu, Hongmei Xu, Zhenxing Shen

**Affiliations:** 1 Shaanxi Key Laboratory of Environmental Monitoring and Forewarning of Trace Pollutions, Shaanxi Environmental Monitoring Center Station, Xi’an 710054, China; xjliutao@stu.xjtu.edu.cn (T.L.);; 2 Department of Environmental Sciences and Engineering, Xi’an Jiaotong University, Xi’an 710049, China; 3 Xi’an Bureau of Ecological Environment Huyi Branch, Xi’an 710003, China; 4 Xi’an Environmental Monitoring Station, Xi’an 710018, China

**Keywords:** volatile organic compounds (VOCs), industrial parks, organized emissions, fugitive emissions, ozone formation potential (OFP), secondary organic aerosol (SOA)

## Abstract

Volatile organic compound (VOC) emissions from industrial parks are a crucial source of urban air pollution. This study assessed VOC emissions and their impact on secondary pollution from three key industries—packaging and printing, pharmaceutical manufacturing, and furniture manufacturing—in a typical industrial park in the Guanzhong region of China. The results revealed considerable variation in organized outlet VOC concentrations between the different industries, with the highest level observed in furniture manufacturing (3449.9 ± 437.6 µg/m^3^) and the lowest level discovered for pharmaceutical manufacturing (410.9 ± 205.5 μg/m^3^). The VOCs were mainly aromatics (40.7%) and alkanes (21.8%), with pentane, isopentane, xylene, and ethylbenzene the most abundant species. Although organized emissions (1151.6 t/y) constituted the primary source of emissions, fugitive emissions (358.1 t/y) remained a major contributor and primarily contributed aromatics and alkanes. Critically, reactivity-based assessment demonstrated that alkenes and aromatics were the principal contributors to the ozone formation potential (>80%). With regard to the secondary organic aerosol formation potential, aromatics were overwhelmingly dominant, accounting for approximately 87% of the total potential, with xylene and ethylbenzene in furniture manufacturing alone contributing 72.9%. The findings highlight the importance of prioritizing controls on highly reactive alkenes and aromatics. Fugitive emission management during storage, mixing, and curing stages should be enhanced and solvents should be substituted to effectively control VOC emissions in industrial parks.

## 1. Introduction

High ambient volatile organic compound (VOC) concentrations remain a global environmental challenge, and regulating VOC emissions from industrial parks has become an increasingly critical challenge [1]. The majority of VOCs are toxic or have an irritating odor and can react with nitrogen oxides (NO_x_) to drive photochemical ozone formation, which is detrimental to the environment [2,3]. The reaction between VOCs and NO_x_ has been identified as the dominant photochemical pathway through which ozone forms in the atmosphere; ozone poses health risks, particularly in terms of its ability to irritate the lungs [4,5]. Consequently, the monitoring and reduction in VOC emissions have garnered increasing attention from governments and the public [6]. The VOC challenge is particularly acute in rapidly industrializing nations such as China. Since 2010, China has implemented a series of stringent policies to control VOC emissions, including the “Action Plan for Air Pollution Prevention and Control.” Nevertheless, observational data reveal that the total anthropogenic VOC emissions nationwide have remained substantial and have increased in several sectors and regions despite the controls [7].

As China undergoes rapid urbanization and industrialization, industrial activity has become the dominant source of VOC emissions, contributing more than half of the national total [8,9]. National industrial emission inventories [10,11] show that since 2000, VOC emissions from industry have increased markedly, whereas emissions from other major sources—such as transportation and household solvent use—have increased more slowly or declined. Industry has thus driven the overall rise in China’s VOC emissions. At the subsector level, inventories for key industries [12] indicate not only high VOC emission intensities but also rapid VOC growth. Sectoral contributions are heterogeneous across provinces and even across parks due to differences in product portfolios, solvent systems, process temperatures, and control technologies. Although volatile chemical products increasingly shape urban VOC budgets [13], solvent use within manufacturing clusters remains a dominant industrial driver. Because ozone chemistry is nonlinear and regime dependent, total VOC mass is an insufficient predictor of air-quality response; speciated, source-resolved data are required to link controls to ozone and secondary organic aerosol (SOA) outcomes [14]. Precisely characterizing the VOC emission characteristics of these key industries and implementing targeted, reactivity-informed controls are essential for meaningfully reducing anthropogenic VOC emissions in China.

Industrial parks are industrial hubs and major sources of high-intensity VOC emissions. VOCs degrade the air quality in areas surrounding these parks and—because several species are toxic, carcinogenic, or strongly irritating—pose substantial health risks to park workers and nearby residents [15,16]. Secondary pollutants formed from the atmospheric oxidation of industrial VOCs, particularly SOA and ozone (O_3_), exert considerable impacts on human beings and the environment in industrialized cities [17,18,19]. Mounting evidence shows that industrial parks exert a substantial effect on their environment, largely through VOC emissions from industrial activities that entail elevated environmental and health risks [20,21,22].

Systematic characterizations of VOC emission profiles and industry-specific emissions within industrial parks in the Guanzhong region have remained scarce. To address this knowledge gap, we selected representative industrial parks in the Guanzhong region and deployed a monitoring network to track in-park pollutants. We performed paired measurements of organized-outlet and fugitive emissions across key sectors, quantified VOC concentrations and speciation through gas chromatography–mass spectrometry (GC-MS) and developed species-resolved VOC profiles. Using these data, we estimated the ozone formation potential (OFP) and secondary-organic-aerosol formation potential, linked emissions to environmental impacts, and apportioned sector- and process-level contributions. The results of this study are expected to provide an integrated, speciated characterization of industrial-park VOCs in Guanzhong, establish a baseline for comparison and trend tracking, and deliver a practical monitoring-and-analysis framework that other industrial parks can adopt for performance evaluation and policy design.

## 2. Materials and Methods

### 2.1. Overview of the Study Area

**Figure 1:**
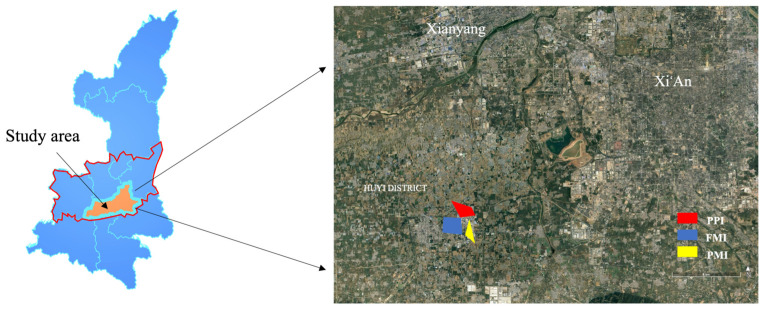
Locations, industrial clusters, and distribution of VOCs observation sites in the industrial park. (In the left map, the blue area represents Shaanxi Province, the red outline denotes the Guanzhong Plain, and the orange area indicates Xi’an City.)

Fengjing Industrial Park in the Huyi District of Xi’an city, shown in Figure 1, was selected as the research site. Located approximately 30 km from Xi’an’s city center, the park is a key industrial cluster within the Guanzhong Plain urban agglomeration. Since its establishment in May 2000, it has gradually developed into a representative provincial-level development zone in southwestern Xi’an, and it plays a major role in promoting regional economic growth and industrial agglomeration. The park, with a planned area of 26 km^2^, has developed a diversified industrial system primarily including packaging and printing industry (PPI), pharmaceutical manufacturing industry (PMI), and furniture manufacturing industry (FMI). Its industrial structure is highly aligned with Xi’an’s industrial layout and regional economic development priorities. Preliminary investigations have indicated that some enterprises within the park emit air pollutants such as VOCs. The park is surrounded by residential areas and villages with a large permanent population, including schools and commercial and residential facilities. Some areas are close to industrial clusters, raising concerns among residents about the surrounding air quality. The various industries in the park result in a complex and diverse set of pollution sources and VOC components, which complicate local air quality management and impact the health of local residents. The park is thus a valuable case for the study of atmospheric pollution in industrial parks.

### 2.2. VOC Sampling and Analysis

VOCs were sampled from both organized and fugitive emission sources within the industrial park. Organized emissions were collected from exhaust stacks and designated outlet points, which are typically part of controlled emission systems designed to capture and treat VOCs before they are released to the atmosphere. Fugitive VOC samples were collected from several locations within the industrial park at which such emissions were most likely to occur. These included storage and mixing areas, where VOCs may escape from open containers or tanks during the handling of volatile substances; production lines, particularly in the packaging and printing and furniture manufacturing sectors, where emissions can be released during material handling, cleaning, or drying; maintenance areas, where leaks or poorly sealed equipment can contribute to VOC releases during repairs or servicing; and curing or drying stations, common in industries such as furniture manufacturing and printing, where solvent-based products can emit VOCs during the heating or drying stages. These sampling sites were selected to provide comprehensive data on fugitive emissions within the industrial park. To ensure data representativeness, VOCs sampling was directly based on continuous and stable monitoring of production operations. Samples were systematically collected during normal manufacturing periods to accurately capture the typical baseline emission profiles. VOCs were collected using Suma canisters (Entech Instruments, Simi Valley, CA, USA). The Suma canisters were cleaned using high-purity nitrogen gas and then humidified to minimize activity adsorption during the cleaning process. The canisters were evacuated (<6.7 Pa), humidified, and bake-cleaned (50 °C, ≥10 cycles) with ultra-high-purity N_2_; field and laboratory blanks were included. Ambient air samples were collected from the 3.2 L Suma canisters with silanized inner walls. Before sampling was performed, the vacuum level of the Suma canister was verified. Subsequently, the canister valve was opened for grab sampling at pre-geolocated points. The collected samples were transferred to the preconcentrator via the autosampler, concentrated in a three-stage cold trap, heated and desorbed, and subsequently analyzed using the GC-MS instrument under its operational conditions [23]. To prevent potential photochemical reactions and sample degradation during collection and transportation, the opaque nature of the Suma canisters naturally blocked light exposure. All collected samples were stored at low temperature in the dark and analyzed within 7 days of collection.

The target compounds were measured using a GC-MS dual detector. A sample was first passed through a preseparation column DB1 (60 m × 0.32 mm × 1.0 µm). This method prevented interference from water molecules and other impurity fragments in the air during the MS detection of low-carbon components. The initial temperature of the GC column box was set at 10 °C and maintained for 3 min. The temperature was then increased to 120 °C at a rate of 5 °C/min and held for 1 min, after which it was increased to 250 °C at 10 °C/min and finally held at 250 °C for 20 min. The column flow rate was set at 1.0 mL/min, and the carrier gas was high-purity helium (purity of >99.999% by volume). The ion source temperature was maintained at 230 °C, the quadrupole temperature at 150 °C, and the transmission line temperature at 250 °C. The electron ionization (EI) (full scan) range was set at 33.0–270.0 u. Target compounds were calibrated with multipoint standards (*R*^2^ > 0.995). The method detection limits ranged from 4 to 81 µg/m^3^, with precision (relative standard deviation, RSD) lower than 5% and recoveries of 85–110%. Coeluting compounds were resolved through MS confirmation. Detection signals for all canister and field blanks were lower than the method detection limit for target species.

To calculate the annual VOC emissions from organized sources, the measured concentrations were converted using the following equation:

$$E=C\times V\times T\times 10^{-9}$$

where *E* is the annual emission (t/y), *C* is the measured VOC concentration (µg/m^3^), *V* is the average stack exhaust flow rate (m^3^/h) obtained from enterprise environmental logs, and *T* represents the annual operating hours (h/y). Furthermore, for data processing, species with concentrations below the method detection limits (MDLs, which ranged from 4 to 81 µg/m^3^) were treated as zero. Uncertainty propagation for emission totals, OFP, and SOAP was estimated considering the variability in measured concentrations and operational parameters.

### 2.3. OFP of VOCs

The OFP is widely used to indicate the photochemical reactivity of VOCs [24,25]. The maximum incremental reactivity (MIR) method is used to determine the OFP of VOCs [26]. The MIR indicates the capacity of various VOC components to engage in atmospheric chemical reactions. The formula is as follows:

$${OFP}_{i}={MIR}_{i}\times {\rho (VOCs)}_{i}$$

where *OFP_i_* represents the OFP of the *i*th VOC component (µg/m^3^); *MIR_i_* is the MIR factor of ozone for the *i*th VOC component, which was determined using available values [27] (g/g); and ρ(*VOC*)*_i_* is the actual observed emission concentration of the *i*th VOC component (µg/m^3^).

### 2.4. Calculation of Secondary Organic Aerosol Formation Potential

The secondary organic aerosol formation potential (SOAP) is often used to evaluate the SOA generation efficiency of specific VOCs [28]. On the basis of a grouped VOC emission inventory, the SOAP value of a specific VOC was calculated as follows:

$${\mathrm{SOAP}}_{i}=Y_{\mathrm{SOA},i}\times {\rho (\mathrm{VOCs})}_{i}$$

where *SOAP_i_* is the SOAP of species *i* (µg/m^3^); *Y_SOA,i_* is the SOA yield of species *i*, with reference to the results of McDonald et al. [13]; and ρ(*VOC*)*_i_* is the actual observed emission concentration of the *i*th VOC component (µg/m^3^).

## 3. Results and Discussion

### 3.1. Characteristics of Organized VOC Emissions in Different Industries

**Figure 2:**
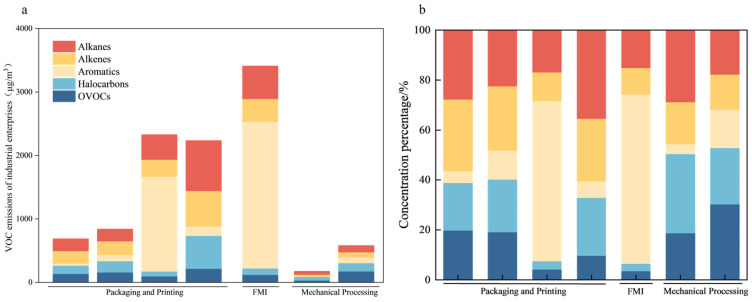
(**a**) Organized outlet VOCs concentrations by industries (µg/m^3^) and (**b**) VOCs class composition (%: aromatics, alkanes, alkenes, halogenated hydrocarbons, OVOCs).

We sampled and analyzed emissions from seven enterprises spanning three industries: the packaging and printing industry (PPI; *n* = 4), pharmaceutical manufacturing industry (PMI; *n* = 2), and furniture manufacturing industry (FMI; *n* = 1). The measured VOC concentrations varied markedly across these sectors (Figure 2a): the FMI had the highest emissions (3449.9 ± 437.6 µg/m^3^), followed by the PPI (1551.5 ± 810.6 µg/m^3^) and PMI (410.9 ± 205.5 µg/m^3^). Figure 2b characterizes the VOC mixture: aromatics were most prevalent (40.7%), followed by alkanes (21.8%), alkenes (16.7%), halogenated hydrocarbons (11.6%), and oxygenated VOCs (9.2%).

The industrial-park VOC concentrations generally exceeded urban background levels, and within the park, PPI facilities typically had higher emissions, driven by high emission factors associated with plastics processing and printing operations [29]. Consistent with this pattern, the PPI enterprises in our study produced VOCs in higher concentrations, and their emissions were rich in pentane, isopentane, C_4_–C_5_ alkenes (butenes and pentenes), xylene, and ethylbenzene, reflecting extensive use of solvent-based inks, cleaners, and varnishes during production. Other studies have reported that gravure printing and laminating rely on solvent-rich materials, with additional diluents such as ethyl acetate and isopropyl alcohol also used; thus, VOC assessment results are often tied to ink and adhesive usage rates [30,31]. Field investigations in the park corroborated that the PPI inks and diluents contained substantial levels of pentane, isopentane, xylene, and ethylbenzene and that UV curing or thermal drying steps generated an appreciable amount of C_4_–C_5_ alkenes. Consequently, selecting lower-volatility solvents and reducing the VOC content of products could be an effective pollution mitigation strategy [32].

**Figure 3:**
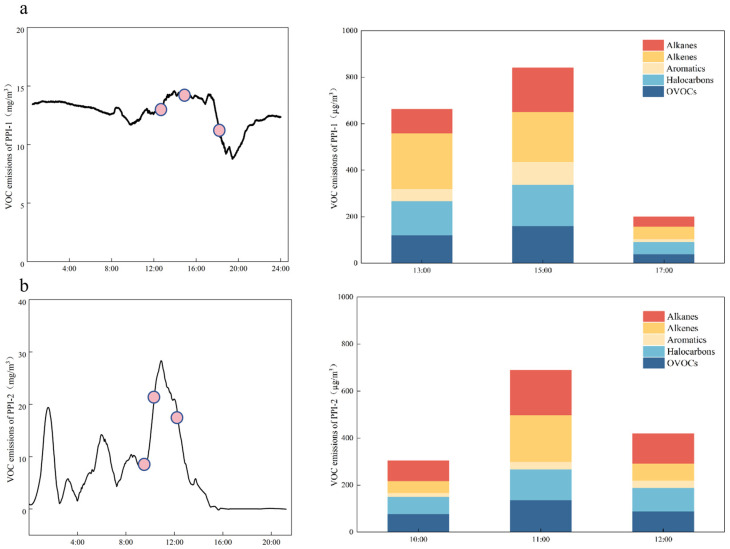
VOCs continuous emission concentrations and time-specific emission characteristics (**a**) PPI-1 (**b**) PPI-2. (The pink circles in the line graphs represent the specific time points when grab samples were taken, which correspond to the bar charts on the right).

The PPI enterprises differed in their time-resolved emission profiles (Figure 3). PPI-1 primarily produced pharmaceutical antiadhesive paper, whereas PPI-2 produced cardboard cartons. PPI-1’s production workshop operated continuously; as shown in Figure 3a, the concentrations of total VOCs produced by this enterprise were relatively stable (11.7 ± 4.2 mg/m^3^), and variability was driven primarily by the alkenes—particularly butenes, pentenes, and related species—associated with the periodic replacement and use of inks and cleaning agents. By contrast, PPI-2 operated intermittently; as illustrated in Figure 3b, the concentrations of its VOC emissions were more variable, reaching up to 27.9 mg/m^3^. Peaks coincided with periods of full-load operation, whereas the compositional profile remained broadly the same over time, indicating that the variability in PPI-2’s emissions was governed mainly by operating intensity rather than shifts in component proportions. These patterns imply distinct control priorities: for continuous lines such as that of PPI-1, substituting lower-alkene solvents in inks and cleaners and tightening fugitive controls during changeovers are likely to preferentially reduce reactivity-weighted emissions. For intermittent lines such as that of PPI-2, load-following abatement and capacity-matched capture systems are likely to yield the largest reductions in mass emissions.

The manufacturers in the FMI used a range of coating technologies—including solvent-based, water-based, and radiation-curable (UV/EB-curable) coatings—and the resulting outlet VOC concentrations differ substantially by coating type; solvent-based coatings generally have the highest concentrations of VOCs [33,34]. In the industrial park investigated in this study, the FMI enterprises had the highest outlet VOC concentrations, consistent with extensive use of solvent-based coatings; emissions were rich in pentane, isopentane, butenes, and 1,1-dichloroethene. Field observations linked these species to specific processes: polyurethane foam blowing agents released pentane and isopentane; curing or hot-pressing of solvent-borne resins generated C_4_ alkenes (butenes); and paint strippers or surface-treatment solvents were the primary sources of 1,1-dichloroethene. The PMI-produced VOCs were dominated by strong odorants; ethanol and methylene chloride are commonly reported in stack emissions and span a wide range of emission levels [35,36]. In our PMI samples, the concentrations of methylcyclohexane, n-decane, dimethylformamide (DMF), and methyl benzoate were comparatively high. Process tracing indicated that extractant and reaction-solvent use accounted for methylcyclohexane and n-decane production; DMF was widely used as a polar solvent; and methyl benzoate appeared as a solvent or reaction product.

### 3.2. Characteristics of Industrial Fugitive Emissions

**Figure 4:**
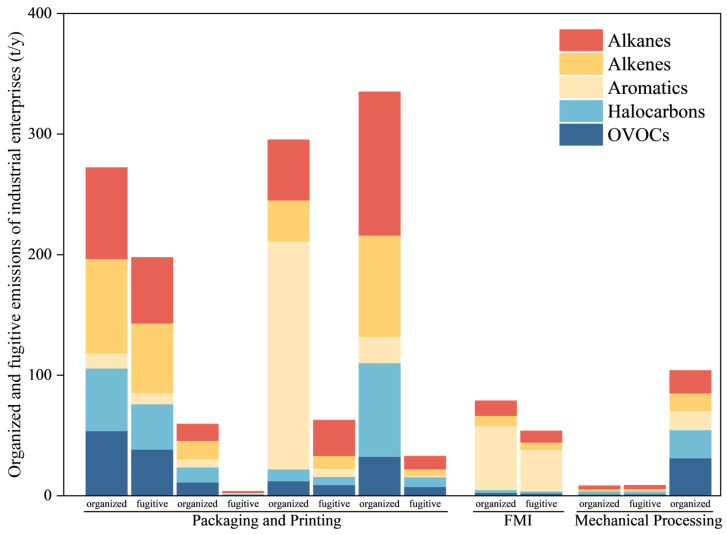
Comparison of organized and fugitive VOCs emissions by industries (t/y).

As shown in Figure 4, the total fugitive VOC emissions were 358.1 t/y. This total was composed of 296.4 t/y from the PPI, 53.7 t/y from the FMI, and 8.5 t/y from the PMI, which corresponded to 82.8%, 15.0%, and 2.4% of the total, respectively. Compared with the organized emissions (1151.6 t/y), the fugitive emissions were substantially lower. Compared with the wide uncertainty ranges from −55% to 88% reported by previous literature [37,38], our assessment of VOC emissions of industrial parks (80.07–122.04%) is reliable. According to field research findings, the primary reason for this was that the enterprises generally adopted effective waste gas collection and treatment measures. Specifically, the capture of VOCs emitted during production processes was maximized through collection systems such as air collection hoods and closed pipelines. Fugitive emissions that may have dissipated were instead centralized as organized emissions and were introduced into efficient end treatment facilities, such as those operating on the basis of thermal or catalytic oxidation and adsorption. Finally, the emissions were organized through a chimney or exhaust cylinder. This approach effectively controlled the amount of fugitive emissions and exhibited superior capture performance relative to central treatment [39].

**Figure 5:**
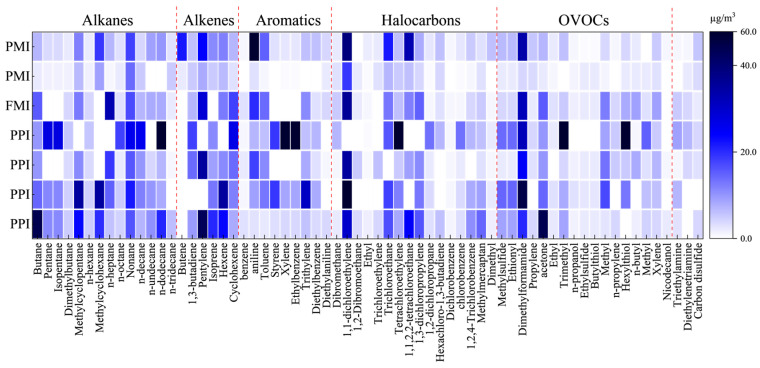
Composition of fugitive VOCs (top species shares, %) with sectoral attribution (PPI, PMI, FMI).

Figure 5 illustrates the composition of the fugitive VOCs. The dominant species were xylene, ethylbenzene, butenes, methylcyclohexane, 1,1-dichloroethene, pentenes, pentane, isopentane, 1,1,2,2-tetrachloroethane, and DMF. Overall, fugitive streams were rich in aromatics and alkanes, and clear intersector differences were discovered. Source attribution indicated that 1,1-dichloroethene, pentenes, and 1,1,2,2-tetrachloroethane were predominantly associated with the PPI, whereas xylene, ethylbenzene, pentane, and isopentane were mainly associated with the FMI. The PPI and FMI contributed substantial amounts of butenes and methylcyclohexane, and DMF contributed a relatively diffuse range of VOCs. These patterns align with operational practices in the respective industries: inadequate sealing of ink storage, open material handling, and low capture efficiency (e.g., an insufficient hood face velocity or incomplete enclosure) increase fugitive emissions before and during printing; larger printing areas further exacerbate emissions [40]. Another study similarly showed that FMI fugitive emissions mirror the composition of raw and auxiliary materials, which are dominated by aromatics [41]. Quantitatively, 84.2%, 79.2%, and 77.3% of fugitive pentene, methylcyclohexane, and 1,1-dichloroethene came from the PPI, respectively; 58.8%, 56.7%, 49.6%, and 46.5% of xylene, ethylbenzene, pentane, and isopentane came from the FMI, respectively.

### 3.3. Contribution to Secondary VOC Pollution and Implications for VOC Control

**Figure 6:**
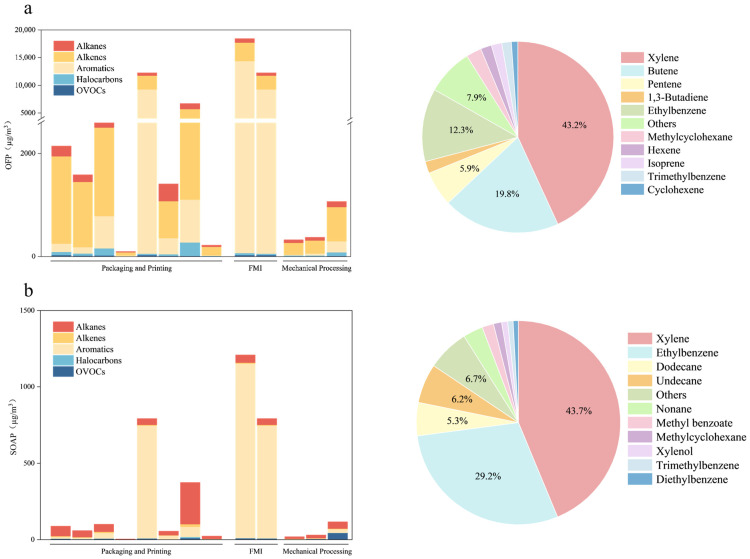
(**a**) Organized and fugitive OFP by industries with top species contributions (µg/m^3^) and (**b**) Organized and fugitive SOAP by industries with top species contributions (µg/m^3^).

To evaluate the OFP in the park, we computed the species-resolved OFP under the MIR framework by using the measured VOC values; the results are displayed in Figure 6. The FMI had the highest OFP (18,429.3 ± 839.3 µg/m^3^), followed by the PPI (5966.3 ± 2470.6 µg/m^3^) and PMI (695.5 ± 277.1 µg/m^3^). In the PPI and PMI (Figure 6a), alkenes and aromatics contributed approximately 62% and 22% of the OFP, respectively. Although the alkene concentrations were lower than the aromatics concentrations, the higher MIR values for alkenes made them the largest contributors to the OFP; thus, controlling alkene emissions should be a primary focus in the PPI and PMI. In the FMI, aromatics and alkenes accounted for approximately 77% and 19% of the OFP, respectively, consistent with the higher aromatic than alkene concentrations reported in Section 3.1; accordingly, aromatic controls are expected to yield the greatest OFP reductions in the FMI. For all sectors, halogenated hydrocarbons constituted a noticeable share of the VOC mixture but contributed little to the OFP because of generally low MIR values.

The most substantial contributors to the OFP were xylene, butenes, pentenes, 1,3-butadiene, ethylbenzene, methylcyclohexane, hexenes, isoprene, trimethylbenzene, and cyclohexene; 1,3-butadiene warrants priority in VOC control due to its toxicity. Xylene contributed the most to the OFP, reflecting both its relatively high MIR and substantial emissions from solvent use in paints, inks, and adhesives across packaging, printing, pharmaceutical, and furniture operations. Alkenes (e.g., butenes, pentenes, hexenes, isoprene, and cyclohexene) also contributed strongly owing to their high MIR values even at low concentrations; sources of alkenes include solvent utilization, fuel volatilization, thermal curing or hot-pressing, and specific reaction steps. To reduce the OFP effectively, differentiated controls should be implemented. These controls should include targeted reductions in highly reactive alkenes in the PPI and PMI (e.g., 1,3-butadiene and C_4_–C_6_ alkenes) and aromatics in the FMI (xylene, ethylbenzene, and trimethylbenzene); they should also include the implementation of capture during mixing, printing, curing, enclosing, and sealing storage or transfer as well as the substitution of low-reactivity, low-volatility solvents.

However, the MIR method evaluates the theoretical maximum ozone formation potential. The actual environmental benefit of reducing these specific VOCs also heavily depends on the local ozone formation regime. Previous studies have demonstrated that the Guanzhong Basin generally exhibits a VOC-limited regime in urban and industrial areas, with recent evidence highlighting a sub-seasonal shift toward transitional or NOx-limited conditions during peak summer meteorological events [42]. Therefore, while prioritizing the control of highly reactive alkenes and aromatics is scientifically justified by our emission profiles, these VOC abatement strategies should be implemented cautiously and tightly integrated with local NO_X_ reduction policies to achieve optimal ambient ozone mitigation.

As indicated in Figure 6b, the FMI had the highest SOAP (1208.7 ± 152.6 µg/m^3^), followed by the PPI (338.6 ± 173.2 µg/m^3^) and PMI (67.8 ± 29.7 µg/m^3^). Aromatics contributed the most to the SOAP in the PPI (~67.3%) and FMI (~93.8%), findings consistent with their high SOA yields. In the PMI, alkanes were dominant, reflecting their large fractional abundance in emissions. The largest contributors were xylene, ethylbenzene, xylenol, trimethylbenzene, diethylbenzene, methyl benzoate, n-undecane, n-dodecane, nonane, and methylcyclohexane; together, xylene and ethylbenzene accounted for 72.9% of the SOAP. This pattern aligns with solvent-use practices: firms in the FMI and PPI rely on solvent-based coatings, inks, and adhesives rich in aromatic solvents (e.g., toluene and xylene), whereas firms in the PMI frequently apply alkane solvents (e.g., n-hexane and cyclohexane), giving their emissions an alkane-rich profile. Mechanistically, aromatics efficiently form low-volatility oxidation products that partition to SOA; although alkanes generally have lower SOA yields per unit mass, their high absolute emissions in the PMI mean that their overall SOAP contribution is dominant. These differences motivate tailored controls: in the FMI and PPI, aromatic solvent use should be reduced, and capture during curing/drying should be enhanced; in the PMI, C_9_–C_12_ alkanes and cycloalkanes emissions should be curbed and lower-SOA-yield solvents should be used.

### 3.4. Emission Reduction Potential and Control Strategies

At the national scale, industrial-source VOC emissions have generally declined over the past decade, with the chemical industry contributing substantially to reductions; nevertheless, considerable abatement potential remains for the paint, coating, and pharmaceutical industries [12]. Consistent with this finding, Figure 3 indicates meaningful room for further reductions across enterprises in our park. For the PPI and FMI, removal efficiency varies widely across processes and species; controlling sources by lowering the VOC content of raw and auxiliary materials remains an effective approach [40,43]. In our dataset, the organized (captured) outlet concentrations exceeded the fugitive levels because facilities generally enclosed their emission points and routed collected streams to end-of-pipe controls. However, the fugitive streams remained rich in aromatics and alkanes, and their OFP burden was nontrivial. As illustrated in Figure 5, alkenes and aromatics dominated the fugitive OFP; in the FMI, the fugitive OFP reached 12,269.6 µg/m^3^, indicating substantial reduction potential. Alkanes and aromatics also had substantial SOAP. These findings highlight the need to prioritize fugitive OFP controls, especially for aromatics and C_4_–C_6_ alkenes, to realize co-benefits for ozone and SOA.

Field inspections identified incomplete enclosure and insufficient capture at ink blending, cleaning, spraying, and drying, leading to persistent fugitive losses. By contrast, the organized and fugitive concentrations from the PMI in this park were low and comparable, reflecting relatively strong controls; further reductions should emphasize fine-grained fugitive management (e.g., enclosure integrity, capture efficiency, and leak detection and repair (LDAR)) rather than major process retrofits.

## 4. Conclusions

This study characterized speciated VOC emissions with high spatiotemporal resolution for a representative industrial park in the Guanzhong region. The VOC concentrations at organized outlets differed markedly between the park’s sectors, with the FMI having the highest concentrations (3449.9 ± 437.6 µg/m^3^), followed by the PPI (1551.5 ± 810.6 µg/m^3^) and PMI (410.9 ± 205.5 µg/m^3^). Aromatics dominated emission mixtures (40.7%), followed by alkanes (21.8%) and alkenes (16.7%); pentane, isopentane, xylene, and ethylbenzene were most abundant VOCs. Although the fugitive concentrations (358.1 t/y) were lower than the organized emission concentrations, patterns persisted, and the fugitive emissions were rich in aromatics and light alkanes and alkenes. Reactivity-based analyses linked emissions to secondary pollution: in the PPI and PMI, alkenes and aromatics contributed the most to the OFP (~62% and ~22%, respectively); in the FMI, aromatics contributed the most to the OFP (~77%). Additionally, in the PPI and FMI, aromatics contributed the most to the SOAP (FMI ~93.8%, with xylene + ethylbenzene ~72.9%); and in the PMI, high levels of C_9_–C_12_ alkanes and cycloalkanes contributed considerably to the SOAP. These results support differentiated controls: prioritize C_4_–C_6_ alkenes (including 1,3-butadiene) in the PPI and PMI and aromatics (xylene, ethylbenzene, trimethylbenzene) in the FMI; strengthen enclosure and capture at mixing, printing, laminating, spraying, curing, and drying stages; implement LDAR, seal storage and transfer processes, and verify hood performance; and use low-MIR, low-volatility solvents and waterborne or UV-curable systems. However, it should be noted that this preliminary study is limited by a small sample size in certain sectors (e.g., FMI). Future studies should incorporate multi-seasonal sampling and local atmospheric photochemical modeling to further validate these findings and optimize region-specific VOC control policies. The present findings provide sector-specific evidence to align park-level-permitting and regional VOC control with ozone and SOA targets. When applying these findings to regional policy, it is important to acknowledge that actual ozone mitigation also depends heavily on the local atmospheric ozone formation regime (e.g., VOC-limited or NOx-limited conditions). Therefore, the reactivity-based controls proposed here should be integrated into a comprehensive, region-specific framework.

## Data Availability

The original contributions presented in this study are included in the article/Supplementary Material. Further inquiries can be directed to the corresponding authors.

## Supplementary Materials

The following supporting information can be downloaded at: https://www.mdpi.com/article/10.3390/toxics14030242/s1, Text S1: Uncertainty analysis [44,45]; Text S2: Detailed description of ambient VOCs sampling and analysis; Figure S1: Schematic diagram of the calculation method for unorganized emissions; Table S1: MDLs of VOCs measured in this study (pptv); Table S2: MIR information used in this study; Table S3: Organized emissions of specified VOCs; Table S4: Fugitive emissions of specified VOCs.
