# Supplementary material for: Organized and Fugitive VOC Emissions from Typical Industrial Parks and Their Impact on Secondary Pollution
Source: Toxics. 2026 Mar 10;14(3):242. doi: 10.3390/toxics14030242 (PMC13030287; doi:10.3390/toxics14030242)
Supplement: Supplementary file 1 [file toxics-14-00242-s001.zip › toxics-4171043-supplementary.pdf]

Supporting Information for

Organized and Fugitive VOC Emissions from Typical Industrial Parks and their Impact on Secondary Pollution

Tao Liu[1,2], Xiaoning Li[3], Weidong Wu[1*], Min Yan[2], Yanxin He[4], Xudong Quan[1], Peng Liu[1], Hongmei Xu[2], Zhenxing Shen[2*]

[1] *Shaanxi Key Laboratory of Environmental Monitoring and Forewarning of Trace Pollutions, Shaanxi Environmental Monitoring Center Station, Xi'an, 710054, China*

[2] *Department of Environmental Sciences and Engineering, Xi'an Jiaotong University, Xi'an, 710049, China*

[3] *Xi 'an Bureau of Ecological Environment Huyi Branch, Xi'an 710003, China*

[4]*Xi'an Environmental Monitoring Station, Xi'an 710018, China*

[*]*Author to whom correspondence should be addressed. E-mail: 18992830293@163.com (Weidong Wu) or zxshen@mail.xjtu.edu.cn (Zhenxing Shen).*

Supplementary captions:

Text S1. Uncertainty analysis.

Text S2. Detailed description of ambient VOCs sampling and analysis.

Figure S1. Schematic diagram of the calculation method for unorganized emissions.

Table S1. MDLs of VOCs measured in this study (pptv).

Table S2. MIR information used in this study.

Table S3. Organized emissions of specified VOCs.

Table S4. Fugitive emissions of specified VOCs.

Text S1. Uncertainty analysis

Monte Carlo simulations have been widely used to assess the uncertainties in the emission estimates [1,2]. This method was also used in the present study to assess the uncertainties in the estimated emissions. A total of 20,000 Monte Carlo simulations were conducted at a 95% coincidence level. Uncertainties in the estimated VOCs emissions are likely associated with the stack exhaust flow rate, annual operating hours, and VOC concentration, which are all assumed to be normally distributed.

Reference:

[1] Zhang B, Shen Z, Sun J, et al. County-level and monthly resolution multi-pollutant emission inventory for residential solid fuel burning in Fenwei Plain, China[J]. Environmental Pollution, 2023, 330.

[2] Wu J, Kong S F, Zeng X, et al. First High-Resolution Emission Inventory of Levoglucosan for Biomass Burning and Non-Biomass Burning Sources in China[J]. Environmental Science & Technology, 2021, 55(3): 1497-1507.

Text S2. Detailed description of ambient VOC sampling and analysis.

Analysis of the VOC samples was conducted by the Institute of Geochemistry, Chinese Academy of Sciences. The air samples were analyzed using a model 7200 preconcentrator (Entech Instruments Inc., California, USA) coupled with an Agilent 5977 gas chromatography–mass selective detector/flame ionization detector (GC-MSD/ FID, Agilent Technologies, USA). In brief, 500-mL air samples were drawn from the canister through a liquid nitrogen-cooled cryogenic trap (0.32 cm × 20 cm) with glass beads (60/80 mesh) at −160 °C. This primary trap was then heated to 10 °C, after which all target compounds were transferred, using pure helium as a mobile phase, to the secondary trap (0.32 cm × 20 cm) at −50 °C with TenaxTA (60/80 mesh) as adsorbents. This micropurge-and-trap step removed most of the redundant H2O and CO2 in the air samples. The secondary trap was then heated to transfer VOCs by helium to a third cryofocus trap (0.08 cm × 5 cm) at −170 °C. Upon completion of this focusing step, the trap was rapidly heated, and the VOCs were transferred to the GC-MSD/FID system. The mixture was first separated using a DB-1 capillary column (60 m × 0.32 mm × 1.0 μm, Agilent Technologies, USA), with helium as the carrier gas, at a constant rate of 4.0 mL/min. Using a splitter, it was then split two ways into a 0.35 m × 0.10 mm I.D. stainless steel line output for mass selective detection and an HP PLOT-Q column (30 m × 1 μm × 0.25 mm, Agilent Technologies, USA) output for flame ionization detection. The GC oven temperature was initially programmed to run at 10 °C for 3 min, after which it was increased to 120 °C at 5 °C/min, 250 °C at 10 °C/min, and was then finally held at 250 °C for 20 min. The MSD operated in a selected ion monitoring mode, and the ionization method was electron impacting (70 eV). A total of 65 VOC species were measured, the minimum detection limits (MDLs) of which are shown in Table S1.

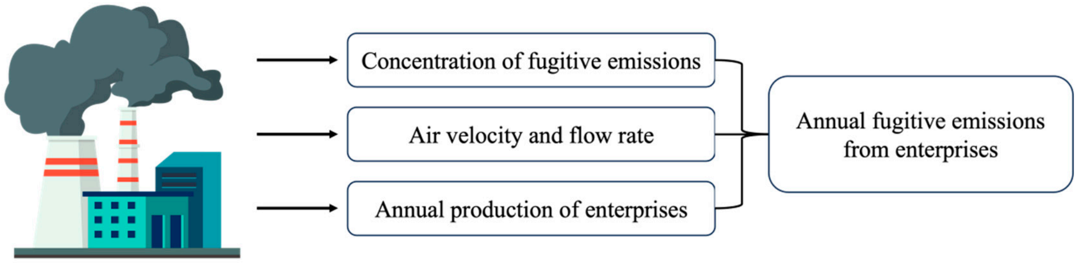

Figure S1. Schematic diagram of the calculation method for unorganized emissions.

Table S1. MDLs of VOCs measured in this study (pptv).

VOC species	MDL	VOC species	MDL
Butane	46	Trichloroethylene	27
Pentane	37	Trichloroethane	20
Isopentane	75	Tetrachloroethylene	12
Dimethylbutane	16	1,1 2,2-Tetrachloroethane	19
Methylcyclopentane	21	1,3-Dichloropropylene	30
n-hexane	42	1,2-Dichloropropane	32
Methylcyclohexane	23	Hexachloro-1,3-butadiene	25
n-heptane	4	Dichlorobenzene	30
n-octane	14	Chlorobenzene	34
Nonane	7	1,2,4-Trichlorobenzene	42
n-decane	11	Dimethylformamide	19
Undecane	20	Acetone	81
n-dodecane	25	Ethyl formate	36
n-tridecane	27	Trimethyl phosphate	18
Butene	40	n-Propanol	39
1,3-Butadiene	46	Methyl tert-butyl ether	30
Pentene	19	n-Propyl acetate	33
Isoprene	20	n-Butyl acetate	42
Hexene	24	Methyl benzoate	17
Cyclohexene	16	Xylenol	26
Benzene	17	n-Decanol	18
Aniline	9	Triethylamine	23
Toluene	24	Diethylenetriamine	47
Styrene	17	Carbon disulfide	74
Xylene	19	Methyl mercaptan	57
Ethylbenzene	23	Dimethyl disulfide	36
Trimethylbenzene	21	Methyl sulfide	19
Diethylbenzene	10	Ethyl mercaptan	29
Diethylaniline	27	Propane mercaptan	35
Methylene bromide	28	Ethyl sulfide	51
1,1-Dichloroethylene	32	Butyl mercaptan	62
1,2-Dibromoethane	32		
Ethyl chloride	28		

Table S2. MIR information used in this study.

VOC species	MIR	VOC species	MIR
Butane	0.94	1,1-Dichloroethylene	0.14
Pentane	1.32	1,2-Dibromoethane	0.27
Isopentane	1.41	Ethyl chloride	0.32
Dimethylbutane	0.98	Trichloroethylene	0.02
Methylcyclopentane	1.71	Trichloroethane	0.05
n-hexane	1.2	1,1 2,2-Tetrachloroethane	1.74
Methylcyclohexane	1.65	1,3-Dichloropropylene	0.47
n-heptane	1.06	Hexachloro-1,3-butadiene	0.33
n-octane	0.87	Dichlorobenzene	0.32
Nonane	0.74	Acetone	0.36
n-decane	0.6	Ethyl formate	1
Undecane	0.5	n-Propanol	1.51
n-dodecane	0.42	Methyl tert-butyl ether	0.62
n-tridecane	0.36	n-Propyl acetate	1.15
Butene	9.73	n-Butyl acetate	1.06
1,3-Butadiene	10.92	n-Decanol	0.6
Pentene	7.21	Carbon disulfide	0.052
Isoprene	10.16	Methyl mercaptan	1.41
Hexene	5.28	Dimethyl disulfide	1.33
Cyclohexene	5.47	Methyl sulfide	3.85
Benzene	0.72	Ethyl mercaptan	1.82
Toluene	4	Propane mercaptan	1.5
Styrene	2.7	Ethyl sulfide	2
Xylene	9.75	Butyl mercaptan	1.4
Ethylbenzene	2.79	Hexanethiol	1.2
Trimethylbenzene	11.66		
Diethylbenzene	7		
Methylene bromide	0.32		

Table S3. Organized emissions of specified VOCs.

No.	Species	PPI-1	PPI-2	PPI-3	PPI-4	FMI	PMI-1	PMI-2
1	Butane	43.995	14.5	10	10	15.5	2	7
2	Pentane	11.19	11.5	95	27.5	147.5	1.5	3.5
3	Isopentane	11.19	11.5	95	27.5	147.5	1.5	3.5
4	Dimethylbutane	4.915	4.5	3.5	5.5	4	1.5	2
5	Methylcyclopentane	23.625	35	11	66.5	12.5	6.5	12
6	n-Hexane	4.915	4.5	3.5	5.5	4	1.5	2
7	Methylcyclohexane	19.92	35	115	343	107	9	19
8	n-Heptane	5.87	15	19	84	33	1	7
9	n-Octane	4.23	5	2	18	3	2	3
10	Nonane	16.34	22	16	26	17	14	18
11	n-Decane	7.16	12	10	26	7	5	4
12	Undecane	10.57	10	4	104	8	0	9
13	n-Dodecane	20.67	8	7	48	8	0	10
14	n-Tridecane	6.1	0	1	0	2	5	3
15	Butene	92.92	84	185	263	295	2	22
16	1,3-Butadiene	9.56	0	14	18	7	5	6
17	Pentene	43.14	72	35	175	29	8	24
18	Isoprene	20.48	13	9	11	0	5	11
19	Hexene	23.625	35	11	66.5	12.5	6.5	12
20	Cyclohexene	8.13	12	14	27	18	3	7
21	Benzene	3.08	1	2	1	3	0	1
22	Aniline	2.79	9	18	7	20	3	49
23	Toluene	2.22	13	11	6	14	1	15
24	Styrene	3.16	19	0	19	0	0	3
25	Xylene	3.85	8.5	725	50.5	1128	0.5	2.5
26	Ethylbenzene	3.85	8.5	725	50.5	1128	0.5	2.5
27	Trimethylbenzene	5.82	31	8	6	11	0	6
28	Diethylbenzene	3.52	8	3	7	3	0	6
29	Diethylaniline	3.73	0	1	0	4	2	4
30	Dibromomethane	2.09	0	1	7	5	3	2
31	1,1-Dichloroethylene	30.21	48	35	207	36	19	39
32	1,2-Dibromoethane	3.56	0	5	0	2	2	0
33	Chloroethane	1.69	0	0.8	0.6	0.9	0.5	1.9
34	Trichloroethylene	3.97	0	6	0	0	3	2
35	Trichloroethane	16.72	18	0	16	13	7	22
36	Tetrachloroethylene	8.33	12	5	55	6	5	6
37	1,1,2,2-Tetrachloroethane	24.67	70	9	105	12	6	33
38	1,3-Dichloropropene	16.12	13	11	86	15	4	8
39	1,2-Dichloropropane	3.11	3.5	2	13.5	1	0.5	2.5
40	Hexachloro-1,3-butadiene	5.98	0	0	7	3	4	6
41	Dichlorobenzene	1.11	1	1	1	1	0	1
42	Chlorobenzene	3.11	3.5	2	13.5	1	0.5	2.5

43	1,2,4-Trichlorobenzene	10.6	8	0	7	4	1	5
44	Dimethylformamide	20.41	45	25	32	32	4	34
45	Acetone	43.995	14.5	10	10	15.5	2	7
46	Ethyl formate	2.29	3	0	2	3	1	2
47	Trimethyl phosphate	8.59	8	4	50	5	0	6
48	n-Propanol	1.27	3	1.5	0	1.7	1.2	0.9
49	Methyl tert-butyl ether	2.56	17.3	13.3	8.8	12.4	1.2	3.7
50	n-Propyl acetate	3.87	0	5	5	4	0	1
51	n-Butyl acetate	1.69	0	8	1	9	2	2
52	Methyl benzoate	4.33	6	3	15	3	2	81
53	Xylenol	3.9	6	7	5	9	3	5
54	n-Decanol	2.16	0	1	0	0	0	1
55	Triethylamine	2.89	7	1	9	5	0	1
56	Diethylenetriamine	0.15	0	4	7	4	2	1
57	Carbon disulfide	3.91	0	3	4	2	3.5	5.5
58	Methanethiol	14.39	9	3	6	5	3	3
59	Dimethyl disulfide	1.61	2	1	2	1	0	6
60	Dimethyl sulfide	8.765	14.5	2	14	4	2	6.5
61	Ethanethiol	8.765	14.5	2	14	4	2	6.5
62	Propanethiol	3.91	0	3	4	2	3.5	5.5
63	Diethyl sulfide	2.125	2.5	2	0	2	2	0.5
64	Butanethiol	2.125	2.5	2	0	2	2	0.5
65	Hexanethiol	4.37	0	3	3	5	3	3

Table S4. Fugitive emissions of specified VOCs.

No.	Species	PPI-1	PPI-2	PPI-3	PPI-4	FMI	PMI-1
1	Butane	25.92	3	14.5	1.5	10	1.5
2	Pentane	6.75	2.5	6	0.5	95	2
3	Isopentane	6.75	2.5	6	0.5	95	2
4	Dimethylbutane	4.12	0	2	1	3.5	1
5	Methylcyclopentane	16.745	0	8	0	11	8.5
6	n-Hexane	4.12	0	2	1	3.5	1
7	Methylcyclohexane	13.74	0	159	5	115	6
8	n-Heptane	3.8	3	6	2	19	2
9	n-Octane	3.76	0	0	2	2	2
10	Nonane	19.59	0	13	9	16	13
11	n-Decane	9.49	0	5	5	10	6
12	Undecane	5.41	0	0	6	4	6
13	n-Dodecane	9.71	0	4	1	7	0
14	n-Tridecane	7.3	0	5	0	1	3
15	Butene	64.76	6	22	1	185	6
16	1,3-Butadiene	12.11	0	17	9	14	0
17	Pentene	30.43	0	25	6	35	11
18	Isoprene	15.97	0	7	0	9	4
19	Hexene	16.745	0	8	0	11	8.5
20	Cyclohexene	5.9	0	4	2	14	6
21	Benzene	3.74	0	1	1	2	1
22	Aniline	2.34	0	0	1	18	1
23	Toluene	2.9	0	1	0	11	1
24	Styrene	1.62	0	0	0	0	0
25	Xylene	3.105	0.5	22	1	725	1
26	Ethylbenzene	3.105	0.5	22	1	725	1
27	Trimethylbenzene	3.5	1	2	0	8	1
28	Diethylbenzene	3.17	0	1	0	3	1
29	Diethylaniline	0.18	5	5	0	1	2
30	Dibromomethane	0	0	7	0	1	1
31	1,1-Dichloroethylene	21.77	10	19	8	35	19
32	1,2-Dibromoethane	2.86	0	0	1	5	0
33	Chloroethane	0	2.2	0	2.1	0.8	0.2
34	Trichloroethylene	8.2	0	0	0	6	0
35	Trichloroethane	16.03	0	0	10	0	12
36	Tetrachloroethylene	8.2	0	0	0	5	0
37	1,1,2,2-Tetrachloroethane	19.27	0	13	4	9	5
38	1,3-Dichloropropene	7.35	0	8	1	11	5
39	1,2-Dichloropropane	1.69	0	1	0	2	1
40	Hexachloro-1,3-butadiene	1.36	0	0	0	0	4
41	Dichlorobenzene	1.41	0	1	0	1	0

42	Chlorobenzene	1.69	0	1	0	2	1
43	1,2,4-Trichlorobenzene	5.59	0	4	1	0	2
44	Dimethylformamide	15.99	0	27	0	25	4
45	Acetone	25.92	3	14.5	1.5	10	1.5
46	Ethyl formate	2.15	0	2	0	0	1
47	Trimethyl phosphate	3.87	0	3	0	4	1
48	n-Propanol	1.73	0	2.1	0	1.5	1.2
49	Methyl tert-butyl ether	1.84	0	5.5	0	13.3	2.1
50	n-Propyl acetate	3.8	0	0	1	5	2
51	n-Butyl acetate	0	1	2	0	8	0
52	Methyl benzoate	3.76	0	4	3	3	6
53	Xylenol	2.08	4	0	3	7	3
54	n-Decanol	0	0	0	1	1	0
55	Triethylamine	2.26	0	0	2	1	0
56	Diethylenetriamine	0	3	0	0	4	0
57	Carbon disulfide	3.33	6.5	1.5	0	3	2.5
58	Methanethiol	16.86	0	3	5	3	3
59	Dimethyl disulfide	0.73	0	0	0	1	0
60	Dimethyl sulfide	3.5	0	0	3	2	2.5
61	Ethanethiol	3.5	0	0	3	2	2.5
62	Propanethiol	3.33	6.5	1.5	0	3	2.5
63	Diethyl sulfide	2.13	0	2.5	1	2	0
64	Butanethiol	2.13	0	2.5	1	2	0
65	Hexanethiol	4.37	0	3	3	5	3
